# Correlation between central corneal thickness and visual field defects, cup to disc ratio and retinal nerve fiber layer thickness in primary open angle glaucoma patients

**DOI:** 10.12669/pjms.331.11623

**Published:** 2017

**Authors:** Muhammad Haroon Sarfraz, Mohammad Asim Mehboob, Rana Intisar ul Haq

**Affiliations:** 1 Dr. Muhammad Haroon Sarfraz, MBBS. Armed Forces Institute of Ophthalmology, Rawalpindi, Pakistan; 2 Dr. Mohammad Asim Mehboob, MBBS. PNS Shifa Naval Hospital Karachi, Pakistan; 3 Dr. Rana Intisar ul Haq, MCPS(Ophth), FCPS(Ophth). Armed Forces Institute of Ophthalmology, Rawalpindi, Pakistan

**Keywords:** Central Corneal Thickness, Cup to Disc Ratio, Layer Thickness, Primary open-angle glaucoma, Retinal Nerve Fibre, Visual field

## Abstract

**Objective:**

To evaluate the correlation between Central Corneal Thickness (CCT) and Visual Field (VF) defect parameters like Mean Deviation (MD) and Pattern Standard Deviation (PSD), Cup-to-Disc Ratio (CDR) and Retinal Nerve Fibre Layer Thickness (RNFL-T) in Primary Open-Angle Glaucoma (POAG) patients.

**Methods:**

This cross sectional study was conducted at Armed Forces Institute of Ophthalmology (AFIO), Rawalpindi from September 2015 to September 2016. Sixty eyes of 30 patients with diagnosed POAG were analysed. Correlation of CCT with other variables was studied.

**Results:**

Mean age of study population was 43.13±7.54 years. Out of 30 patients, 19 (63.33%) were males and 11 (36.67%) were females. Mean CCT, MD, PSD, CDR and RNFL-T of study population was 528.57±25.47µm, -9.11±3.07, 6.93±2.73, 0.63±0.13 and 77.79±10.44µm respectively. There was significant correlation of CCT with MD, PSD and CDR (r=-0.52, p<0.001; r=-0.59, p<0.001;r=-0.41, p=0.001 respectively). The correlation of CCT with RNFL-T was not statistically significant (r=-0.14, p=0.284).

**Conclusion:**

Central corneal thickness had significant correlation with visual field parameters like mean deviation and pattern standard deviation, as well as with cup-to-disc ratio. However, central corneal thickness had no significant relationship with retinal nerve fibre layer thickness.

## INTRODUCTION

Glaucoma is the second leading cause of blindness in the world.^1^ It is an irreversible optic neuropathy that has potential sight threatening consequences. All the epidemiological surveys have concluded that the disease is highly underdiagnosed, and most of the patients remain un-diagnosed till later stages, where advanced damage has already taken place.^2^ All investigations have been aimed at early diagnosis of this condition to warrant early management and control of associated risk factors. Primary Open Angle Glaucoma (POAG) makes the largest percentage of glaucoma patients, and is also the most under-diagnosed condition.^3^ Since there is no secondary cause, except genetic predisposition, the condition is usually asymptomatic, with diagnosis only in late stages when irreversible glaucomatous damage has already ensued.

Doughty was the first one to describe the correlation between Central Corneal Thickness (CCT) and Intraocular Pressure (IOP) measurement.^4^ Ocular Hypertension Study (OHTS) also significantly highlighted the importance of this measurement in overall diagnosis of glaucoma. Multiple studies have highlighted the correlation of CCT with other diagnostic parameters of glaucoma.^5^ The glaucomatous damage is assessed by detection of Visual Field (VF) defects, which shows the glaucomatous damage in functional terms. Humphrey Visual Field (HVF) is an automated perimetry widely used to detect VF defects and monitor glaucoma progression.^6^ The indices like mean deviation (MD) and Pattern Standard Deviation (PSD) show the damage of glaucoma in relation to normal population, and despite their limitations, are widely used in grading and staging of glaucoma.^7^ Cup-to-disc ratio (CDR) is the evaluation of optic disc using slit lamp microscope and fundus viewing lens. It indicates the diameter of the cup expressed as a fraction of the diameter of the disc, and is an important clinical finding in glaucoma suspects and patients.^8^

Several studies have shown its correlation with POAG. With advent of Spectral Domain Optical Coherence Tomography (SD OCT), the diagnosis of glaucoma was revolutionized.^9^ This non-invasive diagnostic facility has been utilized to detect structural glaucomatous damage, which is thought to precede the functional damage. The device measures peripapillary Retinal Nerve Fibre Layer Thickness (RNFL-T), which is believed to be thinned as a result of glaucoma.^10^ No study, has been conducted in Pakistani population to evaluate the correlation between CCT and these important glaucoma indices. Since CCT varies significantly with ethnicity, the results from international literature cannot be ‘generalized’ to our population.

This study has been conducted to evaluate the correlation of CCT with VF parameters like MD and PSD, as well as with CDR, and RNFL-T in Pakistani POAG patients.

## METHODS

After approval by the hospital ethical review committee, informed written consent was taken from the patients prior to inclusion in the study. Keeping significance level of 5% and confidence level of 95%, a sample size of 60 eyes was calculated.^11^ Patients from either gender, aged between 20-60 years, with Best Corrected Visual Acuity (BCVA) of 6/6 on Snellen’s visual acuity chart, diagnosed as POAG on basis of bilateral involvement, IOP > 21mmHg on diagnosis, open anterior chamber, characteristics HVF defects, absence of signs of secondary glaucoma or a non-glaucomatous cause for the optic neuropathy were included. Patients with known history of corneal diseases, trauma, ocular surgery, history of laser surgery (Corneal, Retinal), hereditary causes of RNFL thinning like high myopia, cataract obscuring view of fundus, retinal diseases like diabetes and hypertension, optic disc anomalies like optic disc drusen, papilledema and optic disc pit were excluded. Subjects fulfilling the inclusion criteria underwent ophthalmic examination including uncorrected and BCVA measurement, slit lamp examination for IOP measurement and exclusion of corneal diseases. The patients underwent measurement of CCT using Topcon SP 3000P Specular Microscope (Topcon Corporation, Tokyo, Japan). Three reading were taken and mean calculated for analysis. VF was assessed using HVF Analyser (Haag Streit international perimeter, Octopus 900, Switzerland) with background luminance set at 31.5 asb, Goldmann perimeter stimulus size III, using fast Swedish Interactive Thresholding Algorithm (SITA) without pupil dilation and after correction of near vision. Printouts showing VF result with MD and PSD were collected. After dilating pupils with one drop of 1% Tropicamide, instilled three times, ten minutes apart, CDR was assessed clinically using slit lamp microscope, and fundus viewing 90D lens, and noted in terms of ratio. RNFL-T was measured using SD OCT (3D OCT-1000 Markll, Topcon Co, Tokyo, Japan) and average RNFL-T was obtained by taking average of four quadrants. All investigations/examinations were taken by single researcher to eliminate observer bias. The pre-devised proforma was completed endorsing subject’s demography, ocular examination and investigations findings.

### Statistical Analysis

Data was evaluated and analyzed using Statistical Program for Social Sciences (SPSS) version 17. Mean and Standard deviation was reported for continuous variables (Age, CCT, MD, PSD, CDR, RNFL-T) while frequency and percentage for nominal/ordinal data (gender, laterality of eyes). Shapiro Wilk’s test was used to check normality of data. Post normality testing, Pearson correlation coefficient was calculated to evaluate relationship between CCT and VF parameters (MD, PSD), CDR and peripapillary RNFL-T. A p value of ≤0.001was considered statistically significant.

## RESULTS

A total of 60 eyes of 30 patients were analysed. Mean age of study population was 43.13±7.54 years (Range 27-56 years). Out of 30 patients, 19 (63.33%) were males and 11 (36.67%) were females. Demographic and clinical data of study population is given in Table-I. Correlation of CCT with MD, PSD, CDR and RNFL-T is given in Table-II. There was significant correlation of CCT with MD, PSD and CDR (r=-0.52, p<0.001; r=-0.59, p<0.001; r=-0.41, p=0.001 respectively). The correlation of CCT with RNFL-T was not significant (r=-0.14, p=0.284).

**Table-I:** Demography and Clinical Data of Study population (n=30).

Characteristic	Value
*Age (Years)*	
Mean ± SD	43.13±7.54
Gender	19 /11
(Male/Female)	(63.33%)/(36.67%)
Laterality	30 / 30
Right/Left	(50%)/(50%)
*Glaucoma Duration (Years)*
Mean ± SD	6.86±5.43
*Topical Anti-glaucoma Treatment*
Monotherapy	4 (13.3%)
Duo-therapy	20 (66.7%)
Tripple Therapy	6 (20%)
CCT (µm) Mean ± SD	528.57±25.47
MD Mean ± SD	-9.11±3.07
PSD Mean ± SD	6.93±2.73
CDR Mean ± SD	0.63±0.13
RNFL-T (µm) Mean ± SD	77.79±10.44

**Table-II:** Correlation of CCT with Various Parameters (n=30).

Parameter	Pearson Correlation Co-efficient (r)	p-value
MD	-0.515	<0.001
PSD	-0.599	<0.001
CDR	-0.413	0.001
RNFL-T	-0.141	0.284

## DISCUSSION

CCT is considered an important variable in glaucoma. Despite its effect on IOP measurement, literature has highlighted its impact on glaucoma related factors with controversies. Literature search shows that thin cornea is not associated with POAG or primary angle closure glaucoma (PACG).^12^ Other studies revealed population with thin CCT to be at increased risk of glaucoma development, and thicker corneas having less glaucoma.^13^ Some studies found out that patients with ocular hypertension had thicker corneas, and those with normal tension glaucoma had thin corneas.^14^ The widely acceptable results are from multicentre OHTS which revealed that the risk for development of glaucoma is greater in eyes with low CCT and lower in eyes with higher CCT.^15^ This is probably attributed to under- and over-estimation of IOP due to variations in CCT.

MD and PSD give the estimation of VF defects, and are widely used to interpret, classify and document progression in glaucoma patients. We found negative and significant correlation between CCT and VF parameters like MD and PSD. Studies utilizing multivariate regression models have found negative and significant relation between CCT and VF parameters like MD and PSD.^16^ In another study to find correlation between CCT and severity of glaucoma using Advanced Glaucoma Intervention Study visual field scoring criteria, it was found that score was significantly higher in the thinner CCT eyes, as compared to the thicker CCT eyes. This confirmed CCT as an independent risk factor for glaucomatous VF defects.^17^ Other studies also highlighted significant negative correlation between CCT and VF parameters.^5^,^6^ Same was found in patients with chronic PACG by Hong S et al., who found thin corneas showing more progression in VF defects.^18^ There are studies showing positive correlation between MD and CCT, and negative correlation between CCT and PSD.^11^ One study showed higher level of glaucomatous damage to be associated with 4-μm thinner CCT, without statistical significance. This show CCT to be having no association with VF defects.^19^ Whether CCT is an independent factor for glaucomatous damage or progression remains controversial.

CDR estimation remains an important clinical finding in glaucoma patients. OHTS shows increased risk of glaucomatous damage in patients having high CDR.^15^ We found negative and statistically significant correlation between CCT and CDR, demonstrating thicker corneas to be having less CDR. This is consistent with findings by Kim JM et al who found negative and significant correlation between CCT and CDR, though after multiple regression analysis the correlation appeared to be not statistically significant.^8^ Wangsupadilok and associates also found negative and significant relation between CCT and CDR.^11^ In one study, a significant negative correlation was detected between CDR and CCT(r = 0.102, P < 0.001).^20^ However, there are studies showing no correlation between CCT and vertical CDR.^19^ One study found significant correlation between CCT and CDR in one eye, and not significant relation in other eye.^21^ Wu and co-workers found negative correlation between these two variable, which was not statistically significant.^22^ Thus, it is implied from our study and literature that thinner corneas have high CDR, and vice-versa.

RNFL-T assessment has superseded VF assessment for early diagnosis of glaucoma. It is believed that RNFL-T assessment gives direct evidence of structural damage, before VF defects are elicited.^23^ We did not find statistically significant correlation between CCT and RNFL-T as measured by SD OCT. This is in contrast to findings by Wangsupadilok and Orapiriyakul, who found significant correlation between these two variables.^11^ In study by Mansouri and associates, the CCT had no significant correlation with RNFL-T when measured by OCT. However, when RNFL-T is measured by enhanced corneal compensation algorithms of scanning laser polarimetry, CCT has significant relation with RNFL-T.^16^ However, the relationship between these two variables was also not found significant in another study implying OCT.^24^ In a study on patients with pseudoexfoliation syndrome, CCT was found to have significant relation with RNFL-T.^25^ We thus deduce that better and accurate evaluation of RNFL-T with modern diagnostic modalities like enhanced corneal compensation algorithms of scanning laser polarimetry shows more thinning of RNFL in thin corneas, highlighting more glaucomatous damage. These findings though need further verification by clinical trials.

### Limitations of the study

In our study, ample size was small, measurement of RNFL-T was done by relatively old machine, and patients with ocular hypertension, normal tension glaucoma and PACG were not included. Comparison between sub-types of glaucoma can provide more meaningful analysis in this regard.

## CONCLUSION

The correlation of CCT with different parameters signifying glaucomatous damage shows the importance of this measurement in glaucoma patients. It not only helps in accurate estimation of IOP, but can further give insight into type and stage of glaucomatous damage.
